# Circulating microRNA profile as a potential biomarker for obstructive sleep apnea diagnosis

**DOI:** 10.1038/s41598-019-49940-1

**Published:** 2019-09-17

**Authors:** Fernando Santamaria-Martos, Iván Benítez, Francisco Ortega, Andrea Zapater, Cristina Giron, Lucía Pinilla, Lydia Pascual, Anunciación Cortijo, Mireia Dalmases, Jose M. Fernandez-Real, Ferran Barbé, Manuel Sánchez-de-la-Torre

**Affiliations:** 1 Group of Translational Research in Respiratory Medicine, Hospital Universitari Arnau de Vilanova y Santa Maria, IRB Lleida, Lleida, Spain; 2 Centro de Investigación Biomédica en Red de Enfermedades Respiratorias (CIBERES), Madrid, Spain; 3 Department of Diabetes, Endocrinology and Nutrition, Institut d’Investigació Biomèdica de Girona (IdIBGi), Girona, Spain; 4 CIBER de la Fisiopatología de la Obesidad y la Nutrición (CB06/03) and Instituto de Salud Carlos III, Madrid, Spain

**Keywords:** Diagnostic markers, Respiratory tract diseases

## Abstract

Evaluation of microRNAs (miRNAs) could allow characterization of the obstructive sleep apnea (OSA) and help diagnose it more accurately. We aimed to examine circulating miRNA profiles to establish the differences between non-OSA and OSA patients. Additionally, we aimed to analyse the effect of continuous positive airway pressure (CPAP) treatment on the miRNA profile. This observational, longitudinal study included 230 subjects referred to the Sleep Unit due to suspected OSA. Expression profiling of 188 miRNAs in plasma was performed in 27 subjects by TaqMan-Low-Density-Array. OSA-related miRNAs were selected for validation by RT-qPCR in 203 patients. Prediction models were built to discriminate between non-OSA and OSA: 1) NoSAS-score, 2) differentially expressed miRNAs, and 3) combination of NoSAS-score plus miRNAs. The differentially expressed miRNAs were measured after 6 months of follow-up. From the 14 miRNAs selected for validation, 6 were confirmed to be differentially expressed. The areas under the curve were 0.73 for the NoSAS-score, 0.81 for the miRNAs and 0.86 for the combination. After 6 months of CPAP treatment, miRNA levels in the OSA group seem to approximate to non-OSA levels. A cluster of miRNAs was identified to differentiate between non-OSA and OSA patients. CPAP treatment was associated with changes in the circulating miRNA profile.

## Introduction

Obstructive sleep apnea (OSA) is a prevalent disease that affects approximately 10–17% of the adult population^1,2^. OSA is characterized by repetitive episodes of upper airway collapse during sleep leading to arousal, nocturnal hypoxemia and changes in intrathoracic pressure. Altogether, these events link OSA to the risk of cardiovascular diseases, metabolic disturbances, and cancer and to higher overall mortality mechanisms^3–6^. This association is of special relevance in the young population, in which OSA could have a higher damaging effect, as the observed cancer incidence is increased in young patients^7^ and the impact of OSA on cardiovascular diseases may not be reversible when treatment is delayed^8^. Thus, new methods of early diagnosis of the disease are needed.

Up to 80% of individuals with moderate-to-severe OSA remain undiagnosed^9^. Diagnosis requires overnight recordings, including time- and resource-consuming procedures^9^. High-throughput diagnostic systems may improve the underdiagnoses of the disease and could reduce unnecessary procedures, decreasing the use of healthcare resources.

Currently, the NoSAS score is one of the best-validated tools for screening OSA populations (area under the curve (AUC) 0.74). The NoSAS score performed significantly better than did the STOP-Bang and Berlin scores^10^. NoSAS is based on a questionnaire and does not include any molecular variables that could help with diagnosis. The search for biomarkers that can help physicians not only diagnose OSA but also understand the physiopathology of the disease is a research priority in the field^9,11^. In this context, microRNAs (miRNAs) have emerged as an opportunity in the era of precision medicine for the screening, diagnosis and management of several diseases^12–16^. miRNAs are a class of small non-coding RNAs that negatively regulate gene expression post-transcriptionally by binding to target messenger RNA (mRNA), leading to either degradation or translational repression and protein synthesis^17^. They play a pivotal role in several biological processes, such as stress response, apoptosis, proliferation and differentiation, and many studies suggest that they are deregulated in several diseases, including cardiovascular diseases, metabolic disturbances and cancer^18^. miRNAs are present in body fluids, resistant to degradation and easily and rapidly (8–10 hours) measurable, fulfilling the criteria of an ideal biomarker in an era of evolving precision medicine^19^.

We aimed to examine circulating miRNA profiles to establish the differences between non-OSA and OSA patients and to explore their clinical significance and contribution to disease diagnosis. Moreover, we aimed to evaluate changes in miRNA patterns after 6 months of continuous positive airway pressure (CPAP) treatment.

## Methods

### Study cohort and sample collection

**Figure 1:**
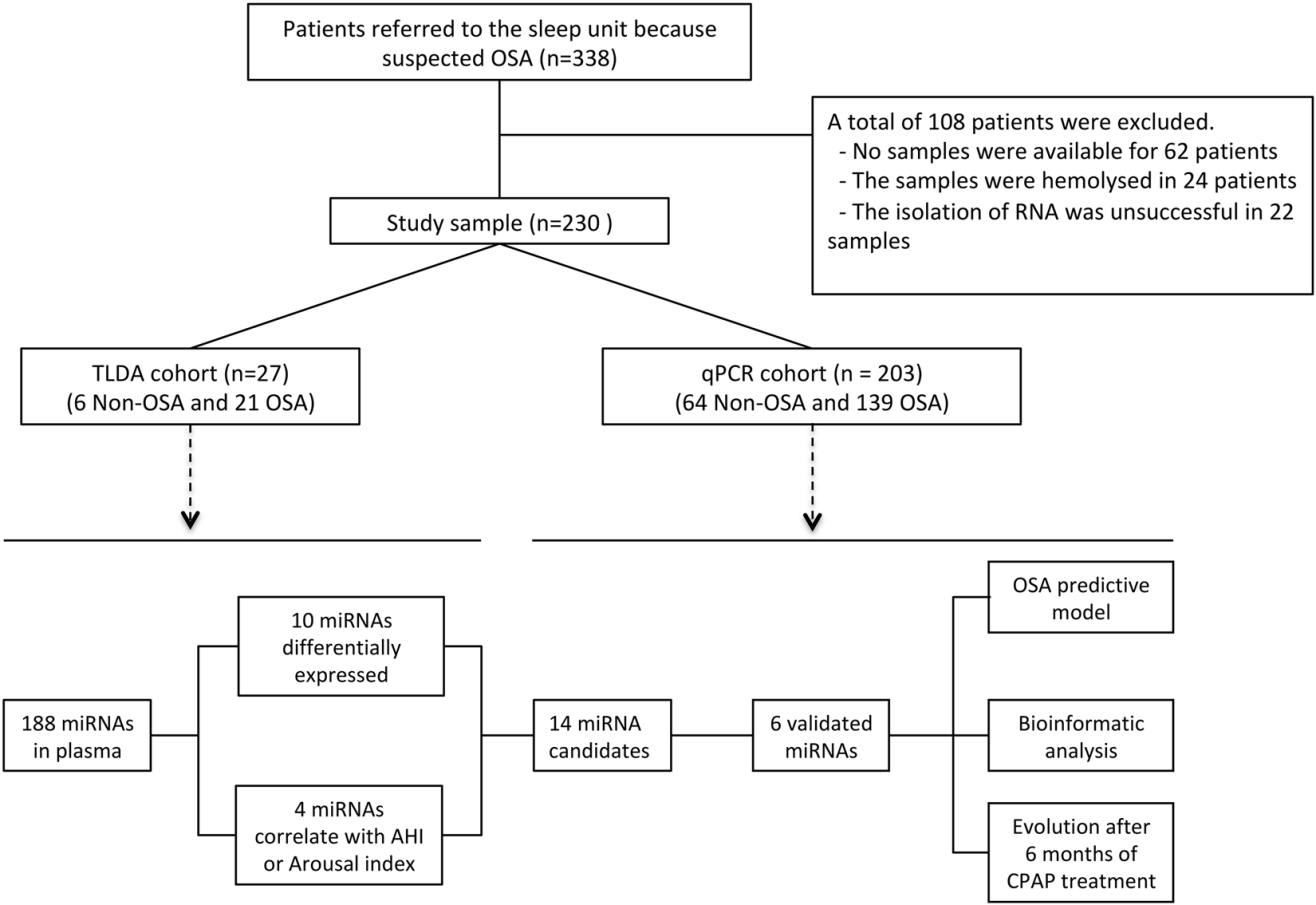
Flowchart of the study. Patients who were referred because of suspected OSA were divided into the TLDA cohort and the qPCR cohort and further divided for study on the basis of non-OSA and OSA. Six non-OSA and 21 OSA patients were used to perform a general screening of 188 miRNAs. Differentially expressed miRNAs were validated in an independent cohort. The analyses in the validation cohort were stratified by sex.

A total of 230 consecutive subjects who were aged between 18 and 60 years, were referred because of suspected OSA and had undergone full polysomnography were enrolled at the Sleep Unit of the Arnau de Vilanova-Santa Maria University Hospital of Lleida (NCT03513926). Subjects were grouped according to their Apnea-Hypopnea Index (AHI) gain into the following: 1) non-OSA group (subjects without moderate-to-severe OSA: AHI <15 events/h), 2) OSA group (moderate to severe OSA: AHI ≥15 events/h). Patients in the TaqMan low-density array (TLDA) cohort were carefully selected. Only patients taking medications related to the most habitual OSA associated pathologies (e.g., hypertension, dyslipidaemia, and cardiovascular events) were not excluded. No patients were excluded from the validation cohort, except those with previous CPAP use or any condition that, in the opinion of the responsible physician investigator, made the person unsuitable for the study (e.g., pregnancy, drug or alcohol consumption, or less than one year of life expectancy). On the other hand, patients without samples or with a haemolysed sample were excluded from the analyses. Additionally, samples with a recovery of RNA less than 1% were excluded (Fig. 1). All recruited patients signed an informed consent form, and the ethics committee of the centre (Clinical Research Ethics Committee of the Arnau de Vilanova-Santa Maria Hospital University Hospital) approved the study. All methods were performed in accordance with current clinical practice guidelines and regulations.

A venous fasting blood sample was obtained from each patient at baseline in the morning immediately after the sleep study between 08:00 and 09:00 a.m. An additional fasting blood sample was obtained at the 6-month follow-up between 08:00 and 09:00 a.m. The blood samples were centrifuged to separate plasma, and all specimens were immediately aliquoted, frozen, and stored in a dedicated −80 °C freezer. No freeze-thaw cycles were performed during the experiment.

### Clinical measurements

All patients underwent full polysomnography at baseline. Apnea was defined as an interruption or reduction in oronasal airflow ≥90% that lasted at least 10 seconds. Hypopnoea was defined as a 30% to 90% reduction in oronasal airflow for at least 10 seconds associated with oxygen desaturation of at least 3% or an arousal on the electroencephalogram. The AHI was defined as the number of apnea and hypopnoea events per hour of sleep. The NoSAS score for screening sleep-disordered breathing was evaluated. The NoSAS score ranges from 0–17 based on neck circumference, body mass index (BMI), age, and gender^10^. All patients were evaluated at baseline and after 6 months of follow-up. Following the National Clinical Guidelines^20^, patients with OSA were treated with CPAP.

### Circulating RNA extraction and purification

RNA was extracted from 300 μL of plasma using a mirVana PARIS isolation kit (Applied Biosystems, Vilnius, Lithuania) according to the manufacturer’s instructions. Non-human cel-miR-39 was spiked into the plasma immediately before extraction. RNA isolation efficiency was examined by RT-qPCR quantification of cel-miR-39 to guarantee homogeneous retrotranscription and cDNA synthesis.

### Circulating miRNA profiling with taqman low density array

To identify plasma miRNA profiles that could help to diagnose OSA, we performed a general screening of 188 circulating miRNAs that have been reported as the most consistent and reliable miRNAs in human plasma samples^21,22^. This circulating profile was evaluated in a cohort of 27 patients (TLDA cohort) that included 6 non-OSA and 21 OSA patients matched by nearest neighbor matching (a procedure to match each OSA patient to a non-OSA subject with the most similar value of the estimated propensity score)^23^ for age and body mass index (Fig. 1). At this point, due to sample size, only male subjects were evaluated. Multiple real-time (RT)-PCRs were performed using a customized TaqMan Low Density Array (TLDA, Life Technologies, Foster City, CA, USA). Briefly, a fixed volume of 3 μL of RNA solution from the 40 μL of RNA isolation eluate was used as the input for the retrotranscription, using a TaqMan MicroRNA Reverse Transcription Kit and TaqMan MicroRNA Multiplex RT Assays, which are customized to run TLDAs. Preamplification was performed using TaqMan PreAmp Master Mix and Megaplex PreAmp Primers for our selected miRNAs. RT-PCR was carried out using an Applied Biosystems QuantStudio™ 7 Flex Real-Time PCR System. Data were processed with the Relative Quantification tool (powered by Thermo Fisher cloud), with a minimal threshold above the baseline (ΔRn = 0.012) and less than 35 thermal cycles (Ct). The results were normalized using a mean-centre normalization method, the gold-standard method when screening a large number of miRNAs^24^.

### Analysis of individual miRNAs

The most reliable and consistent candidates for endogenous control (i.e., hsa-miR-106a and hsa-miR-186) were identified in TLDA analyses and were used together with spike-in (cel-miR-39) for normalization^25^. Then, individual TaqMan hydrolysis probes (Applied Biosystems) were applied to analyse the expression of the set of differentially expressed miRNAs in the qPCR cohort (Fig. 1). Additionally, to assess the evolution of miRNAs after CPAP treatment, we evaluated the changes in miRNA expression of the validated candidates after 6 months of CPAP treatment, comparing non-OSA subjects with OSA patients treated with CPAP.

### Enrichment analysis

The web-based computational tools miRWalk, DIANA-mirPath, TargetScan, DAVID and miRanda were used to predict the target genes and altered pathways of the differentially expressed miRNAs.

### Statistical analysis

To prevent biases due to differential miRNA pattern expression, all analyses were stratified by gender. Comparability between non-OSA and OSA patients was assessed using the Mann-Whitney U test for quantitative characteristics and Fisher’s exact test for qualitative variables. The differences in miRNA expression between groups were evaluated by relative quantification using linear models for arrays (LIMMA)^26^. Spearman’s rank correlation coefficient was used to evaluate the association between miRNA levels and polysomnography parameters. False discovery rate-adjusted p-values were calculated to adjust for the performance of multiple paired comparisons.

Three logistic models were fitted for OSA risk modelling: 1) miRNAs showing differential expression in the validation phase, which were dichotomized by selecting the cut-off point as the point on the receiver operating characteristic (ROC) curve closest to the upper left corner of the unit square and were included in the model; 2) NoSAS score as a continuous variable; and 3) The combination of NoSAS and differentially expressed miRNAs. The Hosmer-Lemeshow test was used to test model calibration. A nonparametric test comparing the AUC of ROC curves^27^ was evaluated. The AUC of the combined model (NoSAS and miRNA) was evaluated in a 10-fold cross-validation. The effect of CPAP on the changes in miRNA expression at the six-month follow-up was estimated by the difference in the mean change between treated OSA patients and non-OSA patients. R software (R Project for Statistical Computing, Vienna, Austria) was used for statistical analysis.

## Results

### Patient characteristics

Of the 338 patients evaluated, 230 were ultimately included (Fig. 1). Patients were mainly middle aged, overweight-obese and male, especially in the OSA group (Table 1). Significant differences were identified in baseline characteristics between non-OSA and OSA groups in age (median 47 vs. 51 years old, p-value = 0.002), BMI (median 27.5 vs. 31.7 kg/m^2^, p-value = 0.015) and sex (68.6% vs. 83.8% male, p-value < 0.001).

**Table 1:** Baseline characteristics of the patients.

	All	Non-OSA (AHI < 15)	OSA (AHI ≥ 15)	p-value
	N = 230	N = 70	N = 160	
Demographic and clinical variables				
Age (years) -median [IQR]-	49.0 [44.0;55.0]	47.0 [40.0;52.0]	51.0 [45.0;55.0]	0.002
Sex (men), n (%)	182 (79.1%)	48 (68.6%)	134 (83.8%)	0.015
BMI (kg/m^2^) -median [IQR]-	30.9 [26.5;34.7]	27.5 [25.4;31.9]	31.7 [28.1;35.2]	<0.001
Systolic blood pressure (mmHg) -median [IQR]-	134 [123;146]	127 [116;137]	136 [127;148]	<0.001
Diastolic blood pressure (mmHg) -median [IQR]-	86.0 [79.5;94.5]	81.8 [75.2;87.8]	88.5 [82.5;95.0]	<0.001
Smoking status: -n(%)-				0.564
Non-smoker	81 (35.5%)	28 (40.0%)	53 (33.5%)	
Former smoker	79 (34.6%)	24 (34.3%)	55 (34.8%)	
Smoker	68 (29.8%)	18 (25.7%)	50 (31.6%)	
Respiratory parameters				
AHI (events/h) -median [IQR]-	29.5 [12.1;51.0]	8.44 [5.00;11.4]	43.8 [27.4;63.9]	<0.001
TSat90 (%) -median [IQR]-	2.40 [0.21;12.7]	0.05 [0.00;0.58]	5.15 [2.00;20.6]	<0.001
Arousal index (events/h) -median [IQR]-	33.8 [21.1;53.6]	18.9 [13.7;25.9]	43.3 [32.1;60.7]	<0.001
Minimum SaO2 (%) -median [IQR]-	82.0 [73.0;87.0]	89.0 [86.0;91.0]	79.0 [71.0;83.0]	<0.001
Mean SaO2 (%) -median [IQR]-	93.0 [91.0;94.0]	94.0 [93.0;95.0]	93.0 [91.0;94.0]	<0.001
ESS (0–24) -median [IQR]-	10.0 [7.00;13.0]	10.0 [7.00;14.0]	10.0 [7.00;13.0]	0.469

Abbreviations: BMI = Body Mass Index; AHI = Apnea-Hypoapnea Index; TSat90 = Night Time with Oxygen Saturation Less Than 90%; ESS = Epworth Sleepiness Scale.

### Identification of plasma miRNAs relevant to OSA

The miRNA profiles were used to identify miRNAs related to OSA in the TLDA cohort (a cohort of men paired by BMI and age). After the analysis, a subset of 14 miRNAs was selected based on an individual p-value < 0.05 when comparing non-OSA and OSA groups and/or a high correlation (r > 0.4) with the AHI or arousal index (Table 2). miR-451, miR-486-3p and miR-133a were selected based on their p-values. On the other hand, miR-181a, hsa-let-7d, miR-199a and miR-199b were selected because of their high correlations with the sleep parameters. Finally, miR-181a-2, miR-495, miR-486, miR-660, miR-345, miR-340 and miR-107 were selected because they met both criteria. From the 14 miRNAs selected, 8 seemed to have less expression in the OSA patients than in the non-OSA patients.

**Table 2:** miRNA candidates that were differentially expressed between non-OSA and OSA male subjects. Fourteen miRNAs were selected based on their fold change and their correlation with the AHI and arousal index.

miRNA	Fold change	p-value	AHI correlation	Arousal index correlation
hsa-miR-181a-2	3.72	0.001	−0.51	−0.42
hsa-miR-495	2.22	0.004	−0.58	−0.79
hsa-miR-451	0.52	0.006	0.18	−0.06
hsa-miR-486	0.52	0.007	0.54	0.28
hsa-miR-660	0.65	0.015	0.42	0.22
hsa-miR-345	0.69	0.021	0.47	0.41
hsa-miR-340	1.51	0.026	−0.61	−0.49
hsa-miR-107	1.62	0.029	−0.63	−0.45
hsa-miR-486-3p	0.55	0.03	0.38	0.31
hsa-miR-133a	0.37	0.049	−0.13	−0.06
hsa-miR-181a	0.73	0.066	0.47	0.5
hsa-let-7d	1.35	0.1	−0.52	−0.67
hsa-miR-199a	1.3	0.157	−0.28	−0.41
hsa-miR-199b	0.81	0.332	0.42	0.36

### Validation of plasma miRNAs relevant to OSA

**Figure 2:**
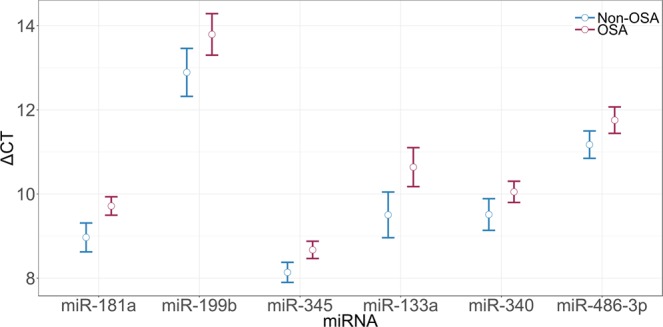
Delta Ct plot means of validated miRNAs (FDR < 0.1).

All potential OSA-related miRNAs selected from TLDA were validated in the qPCR cohort. Among men, the analysis confirmed that the circulating concentrations of hsa-miR-181a, hsa-miR-199b, hsa-miR-345, hsa-miR-133a, hsa-miR-340 and hsa-miR-486-3p were decreased in the OSA group after adjustment for BMI and age (Table 3 and Fig. 2). Of these, miR-181a seemed to be correlated with the AHI (rho = 0.29) and with the arousal index (rho = 0.25) and miR-345 with the AHI (rho = 0.21). To further explore the results, mild OSA patients were removed in order to confidently report the results between non-OSA (AHI <5 events/h) and OSA (AHI ≥15 events/h) subjects. This analysis reported increased differences between non-OSA and OSA subjects (except for miR-199b and miR-486-3p) (see e-Table 3).

**Table 3:** Validation of miRNA candidates. Six miRNAs were found to be differentially expressed between non-OSA and OSA patients. P-values adjusted for age and BMI.

miRNA	Fold change	p-value	FDR correction
hsa-miR-181a	0.59	0.001	0.013
hsa-miR-199b	0.42	0.008	0.056
hsa-miR-345	0.71	0.014	0.056
hsa-miR-133a	0.47	0.016	0.056
hsa-miR-340	0.67	0.029	0.072
hsa-miR-486-3p	0.64	0.031	0.072
hsa-miR-181a2	0.51	0.066	0.133
hsa-miR-199a	0.54	0.184	0.318
hsa-miR-660	0.79	0.214	0.318
hsa-miR-451	0.82	0.227	0.318
hsa-let-7d	0.93	0.591	0.752
hsa-miR-486	1.06	0.665	0.776
hsa-miR-107	1.07	0.817	0.832
hsa-miR-495	0.93	0.832	0.832

FDR <0.1 was considered statistically significant.

Among women, only hsa-miR-451 (fold change = 1.6 and p-value = 0.057) seemed to be differentially expressed after adjustment for BMI and age, but this significance is lost after FDR correction (corrected p-value = 0.54) (see e-Table 4).

### Prediction model

**Figure 3:**
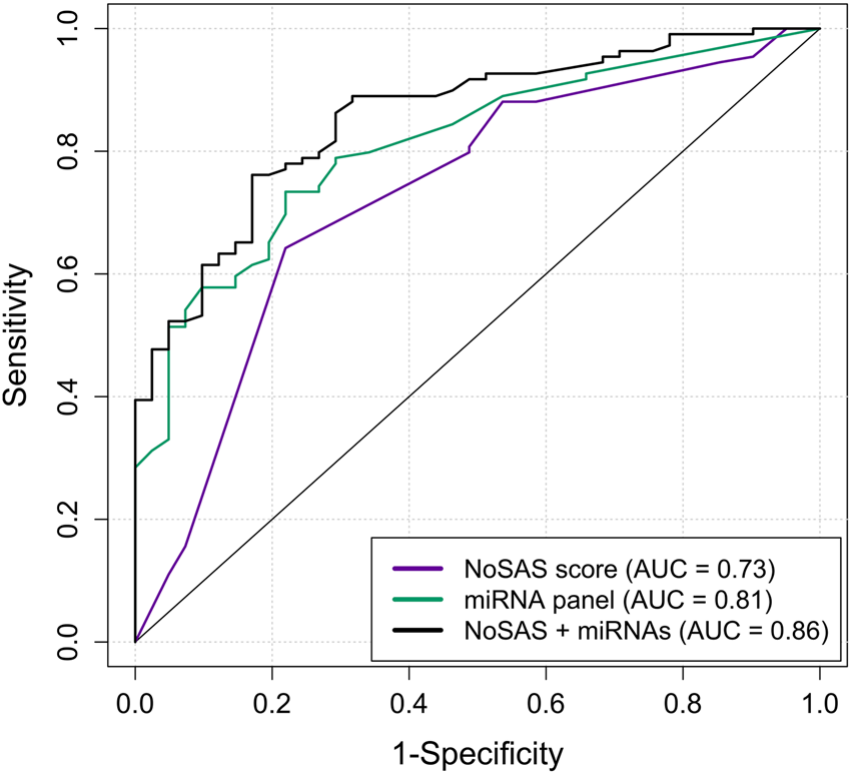
Discriminatory ROC plot of the models for screening OSA using (**A**) NoSAS; (**B**) 6 miRNAs; and (**C**) a combination of NoSAS and miRNAs.

Logistic regression models were generated for male patients as a tool to differentiate between non-OSA and OSA patients. Three multivariate models were fitted to classify the patients: A) the NoSAS score; B) the validated miRNAs and C) a combination of NoSAS score and validated miRNAs (Fig. 3). All the models exhibited good calibration and discrimination. The AUCs were 0.73 for the NoSAS score, 0.81 for the miRNAs and 0.86 for the combination. A comparison of the models based on AUC showed that the combination of NoSAS score and miRNAs was the best model for discriminating OSA patients (Table 4). The AUC of the combined model showed similar results in cross-validation (AUC = 0.83).

**Table 4:** Comparison of ROC models. A) NoSAS scores, B) miRNA model, C) combination.

Comparison	AUC difference	p-value	Correlation
A vs. B	−0.08	0.16	0.01
A vs. C	−0.13	0.002	0.54
B vs. C	−0.04	0.065	0.76

### Changes in validated plasma miRNAs after CPAP treatment

We evaluated the miRNAs that were differentially expressed and validated, at baseline and after 6 months of follow-up. We found that in OSA CPAP-treated patients (median adherence 5.24 h/night), miRNA levels seem to increase, except for miR-486-3p, compared to those in non-OSA patients. Despite this observation, only changes in miR-345 reached statistical significance (Table 5).

**Table 5:** Post-pre mean differences in differentially expressed miRNAs between non-OSA and OSA patients after 6 months of CPAP treatment.

miRNA	Estimate difference	Lower bound	Upper bound	p-value
hsa-miR-181a	−0.514	−1.524	0.495	0.314
hsa-miR-199b	−0.372	−1.714	0.971	0.581
hsa-miR-345	−1.419	−2.416	−0.423	0.006
hsa-miR-133a	−0.216	−1.443	1.012	0.728
hsa-miR-340	−0.418	−1.477	0.642	0.435
hsa-miR-486-3p	0.550	−0.567	1.668	0.330

### Bioinformatic analysis

The enrichment analysis, including the 6 miRNAs confirmed as differentially expressed between non-OSA and OSA patients at baseline, revealed 69 dysregulated pathways. A total of 1,743 genes were identified to be deregulated; 352 genes were targeted by miR-133a, 188 genes by miR-181, 167 genes by miR-199b, 47 genes by miR-340, 185 genes by miR-345, and 804 genes by miR-486-3p. Of the 1,743 predicted targeted genes, only 626 were unique genes, and IGF1R, KRAS, RUNX1T1 and SLC9A8 were regulated by 3 miRNAs.The most frequently enriched pathways were categorized under biological processes connected with cancer (renal cell carcinoma, melanoma, etc.), cardiovascular diseases (dilated cardiomyopathy, hypertrophic cardiomyopathy, etc.), and signalling (sphingolipid signalling, adrenergic signalling of cardiomyocytes, etc.) (see e-Table 6 and e-Fig. 6).

## Discussion

A singular male-specific cluster of miRNAs functionally associated with sleep, cardiovascular diseases, metabolic disorders and cancer significantly contributes to the discrimination between non-OSA and OSA patients in the sleep unit. The present study also showed that CPAP use is associated with changes in the miRNA profile that could influence the overall risk of suffering OSA-related diseases.

Circulating concentrations of 6 miRNAs were significantly reduced in OSA subjects compared with those in non-OSA subjects. We also investigated the discriminatory ability of the miRNAs. We compared the NoSAS score, miRNAs and a combination of both. The NoSAS score AUC was consistent with previous results, with an AUC of 0.73, but lower than that obtained with miRNAs (AUC = 0.81)^10,28^. The correlation between both AUCs is very low (0.01), which could be argued because NoSAS and miRNAs could explain different components of OSA, resulting in a higher AUC of 0.86 for the combination of both, which could represent a very powerful tool that combines anthropometric and molecular variables to explain OSA variability. Finally, the longitudinal study showed that after CPAP treatment, miRNAs seem to increase their circulating concentration, at least partially recovering the non-OSA phenotype. Decreased blood pressure could have contributed to the miRNA changes detected after CPAP treatment. In fact, as previously reported by our group, CPAP treatment changes the miRNA profile, and these changes correlate with blood pressure changes that occur following CPAP treatment. Additionally, these changes are associated with the response to treatment of the patient^14^.

This study identifies the utility of miRNAs as biomarkers that could contribute to the personalized management of OSA in the sleep unit. Recent studies have demonstrated the importance of miRNAs in the control of many processes in health and disease^14,19,21^. To the best of our knowledge, this is one of the first studies to use miRNAs to identify a signature and characterize OSA in patients who are referred to a sleep unit. A similar study was conducted^29^, in which the researchers identified that in a preliminary phase, 104 miRNAs were differentially expressed in serum between OSA and non-OSA subjects. Of these miRNAs, only 4 were validated by qPCR. We also attempted to validate miR-107 and miR-199 in our study. In both studies, miR-199 was downregulated when comparing OSA patients vs. non-OSA subjects; however, in our study, miR-107 was not confirmed to be differentially expressed. These possible discrepancies could be due to the different sample types utilized (plasma vs. serum) and our different normalization methods (endogenous control + spike-in vs. spike-in). Additionally, the data reported in this previous study did not seem to be adjusted by confounding variables.

A total of 6 miRNAs have been identified to be differentially expressed between non-OSA and OSA patients. This set of miRNAs seems to have the ability to differentiate between non-OSA and OSA patients, which is effective and practical for clinical use and enables the identification of patients with OSA. Recently, a seminal study developed by our group identified miRNAs as a useful tool in the management of patients with OSA and resistant hypertension^14^. In this study, we demonstrated the utility of miRNAs by identifying a singular cluster of miRNAs (HIPARCO-Score) that identifies those patients who will have a favourable blood pressure response to CPAP treatment. The present study corroborates the clinical usefulness of miRNAs for the diagnosis of OSA at sleep unit level.

The set of 6 miRNAs that are differentially expressed between non-OSA and OSA patients is related to relevant canonical pathways. First, we identified that miRNAs are misregulating pathways related to cardiovascular diseases, specifically several cardiomyopathies, in concordance with the hypothesis of a difference in cardiovascular risk between non-OSA and OSA patients. Second, identifying the molecular pathways related to both cancer and OSA is one of the hallmarks of OSA research. Additionally, an early diagnosis of OSA could be especially important because the association of OSA with cancer incidence is limited to young people^7^. Several studies have shown the association of OSA with several types of cancer, especially in young patients^30^. In the present study, we identified several cancer-related pathways that could be altered in patients with OSA, and some of the altered pathways are related to melanoma. These results are in accordance with previous literature in which the impact of OSA on melanoma and some of the intermediate pathways have been addressed^31,32^. Similar results were found in a previous work^25^, where bioinformatic analyses showed that OSA could alter cardiovascular and cancer-related pathways.

Specifically, these miRNAs play an important role in some pathways related to common diseases associated with OSA. OSA is well known to be related to deregulated nutrient sensing, especially glucose. Patients with OSA are more likely to develop insulin resistance due to oxidative stress and a lower concentration of oxygen in pancreatic ß-cells^33^. Recently, miR-486 has been associated with the HOMA index and fasting levels of glucose and insulin^34^. Moreover, miR-340 seems to deregulate IGF1R gene expression, as shown in bioinformatic analyses. Previous studies observed associations between OSA and melanoma. Additionally, miR-340 is known to regulate MITF gene expression, which is a gene that plays a key role in melanocyte development. Recently, one study found that miR-340 regulates RAS–RAF–mitogen activated protein kinase (MAPK) signalling by modulating the expression of multiple components of this pathway^35^, and this association could explain, at least in part, the relationship between OSA and melanoma. Several genes regulated by these miRNAs are involved in cardiovascular diseases. miR-133a regulates cardiomyocyte proliferation and suppresses smooth muscle gene expression in the heart^36^ and is dysregulated in human myocardial infarction^37^. Finally, miR-199b is involved in the response to the hypoxia-repressing HIF1A gene^38^. HIF1A is a key gene in the response to hypoxic stress, and its expression is deregulated in OSA patients^39^. Moreover, IGF1R, KRAS, RUNX1T1 and SLC9A8 are regulated by 3 miRNAs. All these genes are related to melanoma pathways and play an important role in the transformation of melanocytes. Recently, one study assessed the molecular relationship between OSA and melanoma, concluding that OSA increased circulating VCAM-1 levels in melanoma patients^31^. This relationship could be an indirect consequence of IGF1R, KRAS, RUNX1T1 and SLC9A8 regulation.

Notably, all miRNAs validated in the present study were downregulated in patients with OSA. As previously reported, chronic hypoxia impairs Dicer (DICER1) expression and activity, resulting in global downregulation of miRNA expression^40^.

Diagnostic and personalized therapeutic decision-making tools are needed to manage OSA, especially in the young population^9,10,41^. Together, these results suggest that the use of miRNAs in OSA management could be a powerful tool for the diagnosis of OSA and to establish the benefits of the treatment for this complex chronic disease.

The present study has several limitations that deserve comment. First, only patients between 18 and 60 years old were studied, and larger studies should be performed to determine the validity of this tool in patients of other ages. The validation showed poor results in women, reflecting the different physiopathology of OSA between women and men. Recent studies have shown that miRNAs have sex-dimorphic expression patterns^42^. Because of the ages of the subjects, oestrogens could play a very important role in these expression patterns. As previously reported, sex-related differences may cause abnormal miRNA expression, and for this reason, stratified analyses are recommended. Further studies are needed to evaluate the whole profile of miRNAs in women with OSA. Despite, RNA isolation efficiency was measured with spike-in cel-miR-39 to guarantee homogeneous retrotranscription and cDNA synthesis; no data about RNA integrity or quantity are available. Although we performed a quantification of a large number of miRNAs (a total of 188 miRNAs) that were previously identified as the most stable miRNAs in plasma and were quantified in at least 70% of the subjects^21,22^, we did not perform whole miRNA quantification. This approximation permits us to use gold standard technology and enables an accurate quantification in a short period of time, with the highest dynamic range^43^. By last, this tool is not able to distinguish mild OSA patients from non-OSA subjects; further studies including a higher number of patients with AHI <5 events/h is required. The strengths of this study reside in a large number of patients who were analysed in two different sets for the TLDA and qPCR cohorts, which permitted us to detect high-magnitude associations. Additionally, some of the basal associations were modified after CPAP use, confirming the importance of these miRNAs in OSA. Finally, all patients were recruited from the sleep unit, performing gold-standard methods for OSA diagnosis (polysomnography) and for miRNA measurement (RT-qPCR).

A singular male-specific cluster of miRNAs was identified that specifically differentiates between non-OSA and OSA patients. This miRNA model, which improves the discriminatory ability, is based in anthropometric and clinical variables. Finally, the use of CPAP treatment was associated with changes in the circulating miRNA profile that partly recovered the non-OSA phenotype.

## Supplementary information

Supplementary information
