# Supplementary material for: Circulating microRNA profile as a potential biomarker for obstructive sleep apnea diagnosis
Source: Sci Rep. 2019 Sep 17;9:13456. doi: 10.1038/s41598-019-49940-1 (PMC6748919; doi:10.1038/s41598-019-49940-1)

**Circulating microRNA profile as a potential**

**biomarker for obstructive sleep apnea diagnosis**

**Authors:** F. Santamaria-Martos^1^, I. Benítez^1,4^, F. Ortega^3,4^, A. Zapater^1^, C. Giron^1^, L. Pinilla^1^, L. Pascual^1^, A. Cortijo^1^, M. Dalmases^1^, J.M. Fernandez-Real^3,4^, F. Barbé^1,2^, M. Sánchez-de-la-Torre^1,2,*^.

**Affiliations:**

^1^Group of Translational Research in Respiratory Medicine, Hospital Universitari Arnau de Vilanova y Santa Maria, IRB Lleida, Lleida, Spain;

^2^Centro de Investigación Biomédica en Red de Enfermedades Respiratorias (CIBERES), Madrid, Spain.

^3^Department of Diabetes, Endocrinology and Nutrition, Institut d'Investigació Biomèdica de Girona (IdIBGi), Girona, Spain;

^4^CIBER de la Fisiopatología de la Obesidad y la Nutrición (CB06/03) and Instituto de Salud Carlos III, Madrid, Spain;

*Correspondence to:

Manuel Sánchez-de-la-Torre, PhD, Hospital Arnau de Vilanova-Santa María, IRBLleida, CIBERES, Avda. Rovira Roure 80, 25198, Lleida, Spain; e-mail: sanchezdelatorre@gmail.com

1. TaqMan Low Density Array determinations

1.1. Baseline characteristics of the cohort

1.2. Quality control

1.2.1. Number of determinations/missings

1.2.2. Ct distribution of miRNAs

1.3. miRNA normalization

2. qPCR determinations

2.1. Baseline characteristics of the cohort

2.2. Quality control

2.2.1. Number of determinations/missings

2.2.2. Ct distribution of miRNAs

2.3. miRNA validation and models

2.3.1.

2.3.2. Validation of differentially expressed miRNAs in women

2.3.3. Univariate logistic regression analysis

2.3.4. Bioinformatic analysis of differentially expressed miRNAs

2.3.5. Main biological processes regulated by the differentially expressed miRNAs

**1. TaqMan Low Density Array determinations**

1.1. Baseline characteristics of the cohort.

e-Table 1: Baseline characteristics of the cohort.

|  | All | Non-OSA (AHI<15) | OSA (AHI ≥15) | p value |
| --- | --- | --- | --- | --- |
|  | N=27 | N=6 | N=21 |  |
|  |  |  |  |  |
| Demografic and Clinical variables |  |  |  |  |
| Age (years) -median [IQR]- | 49.0 [41.0;55.0] | 43.5 [39.2;47.8] | 51.0 [44.0;56.0] | 0.115 |
| Sex (men), n (%) | 27 (100%) | 6 (100%) | 21 (100%) |  |
| BMI (kg/m2) -median [IQR]- | 27.2 [25.4;30.4] | 25.1 [23.1;29.3] | 27.2 [26.2;30.5] | 0.180 |
| Smoking status: -n(%)- |  |  |  | 0.723 |
| Non smoker | 8 (29.6%) | 1 (16.7%) | 7 (33.3%) |  |
| Former smoker | 12 (44.4%) | 3 (50.0%) | 9 (42.9%) |  |
| Smoker | 7 (25.9%) | 2 (33.3%) | 5 (23.8%) |  |
|  |  |  |  |  |
| Respiratory parameters |  |  |  |  |
| AHI (events/h) -median [IQR]- | 29.7 [18.3;42.7] | 7.36 [6.18;10.4] | 32.4 [28.1;49.2] | <0.001 |
| TSat90 (%) -median [IQR]- | 0.60 [0.22;6.70] | 0.03 [0.00;0.09] | 2.28 [0.33;8.37] | 0.002 |
| Arousal index (events/h) -median [IQR]- | 36.8 (17.0) | 15.9 (10.1) | 42.8 (13.6) | <0.001 |
| ESS (0-24) -median [IQR]- | 9.96 (5.59) | 12.3 (7.09) | 9.21 (5.03) | 0.351 |
| Abbreviations: BMI = Body Mass Index; AHI = Apnoea-Hypoapnoea Index; TSat90 = nighttime with oxygen saturation less than 90%; ESS= Epworth Sleepiness Scale. | | | | |

1.2. Quality control

e-Figure 1: Number of determinations/missings.

Samples

e-Figure 2: Raw Ct distribution of miRNAs.


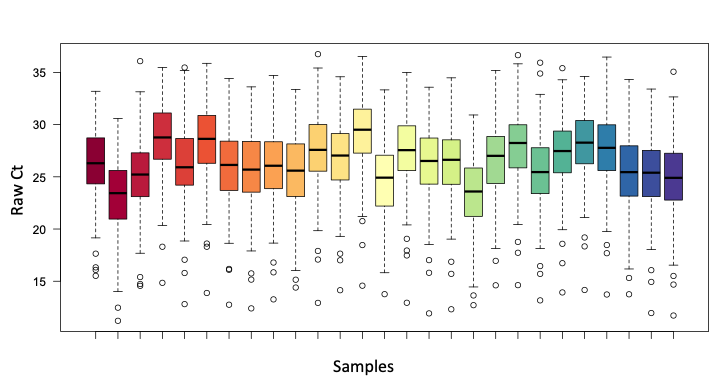


1.3. miRNA normalization.

e-Figure 3: Density plot of miRNA normalization. A) density of raw Cts of each subject. B) Mean-center normalized density plot.


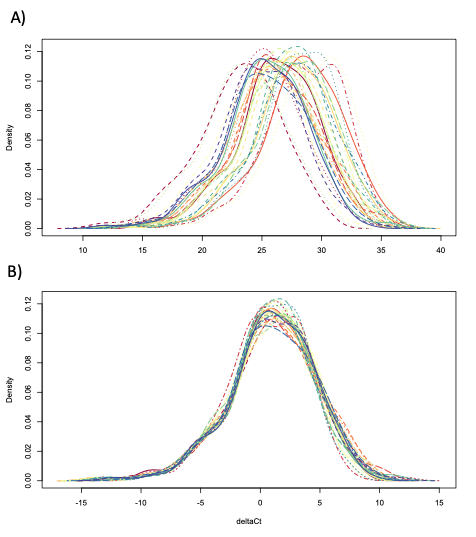


**2. qPCR determinations**

2.1. Baseline characteristics of the cohort

e-Table 2: Baseline characteristics of the cohort

|  |  | Men | | |  | Women | | |
| --- | --- | --- | --- | --- | --- | --- | --- | --- |
|  |  | Non-OSA (AHI<15) | OSA (AHI ≥15) | p value |  | Non-OSA (AHI<15) | OSA (AHI ≥15) | p value |
|  |  | N=42 | N=113 |  |  | N=22 | N=26 |  |
|  |  |  |  |  |  |  |  |  |
| Demografic and Clinical variables |  |  |  |  |  |  |  |  |
| Age (years) -median [IQR]- |  | 47.0 [41.2;52.0] | 49.0 [44.0;55.0] | 0.142 |  | 45.5 [38.5;53.8] | 53.5 [49.2;57.0] | 0.001 |
| BMI (kg/m2) -median [IQR]- |  | 27.1 [25.6;29.6] | 32.5 [29.1;35.7] | <0.001 |  | 29.1 [24.1;34.7] | 33.3 [28.5;35.8] | 0.153 |
| Smoking status: -n(%)- |  |  |  | 0.281 |  |  |  | 0.793 |
| Non smoker |  | 16 (38.1%) | 31 (27.7%) |  |  | 11 (50.0%) | 15 (60.0%) |  |
| Former smoker |  | 15 (35.7%) | 41 (36.6%) |  |  | 5 (22.7%) | 5 (20.0%) |  |
| Smoker |  | 11 (26.2%) | 40 (35.7%) |  |  | 6 (27.3%) | 5 (20.0%) |  |
|  |  |  |  |  |  |  |  |  |
| Respiratory parameters |  |  |  |  |  |  |  |  |
| AHI (events/h) -median [IQR]- |  | 9.29 [5.65;12.0] | 45.6 [29.4;64.6] | <0.001 |  | 5.09 [2.88;9.17] | 39.3 [23.3;66.5] | <0.001 |
| TSat90 (%) -median [IQR]- |  | 0.12 [0.00;0.60] | 5.40 [2.30;21.3] | <0.001 |  | 0.02 [0.00;0.29] | 6.43 [2.08;20.7] | <0.001 |
| Arousal index (events/h) -median [IQR]- |  | 19.0 [13.6;25.7] | 45.3 [33.1;62.4] | <0.001 |  | 15.4 [14.2;26.8] | 36.2 [23.7;58.4] | <0.001 |
| ESS (0-24) -median [IQR]- |  | 9.00 [7.00;12.0] | 10.0 [6.50;13.0] | 0.553 |  | 11.5 [9.0;15.0] | 11.0 [7.0;15.0] | 0.453 |
| Abbreviations: BMI = Body Mass Index; AHI = Apnoea-Hypoapnoea Index; TSat90 = nighttime with oxygen saturation less than 90%; ESS= Epworth Sleepiness Scale. | | | | | | | | |

2.2. Quality control

e-Figure 4: Number of determinations/missings.

Samples

e-Figure 5: Raw Ct distribution of miRNAs.


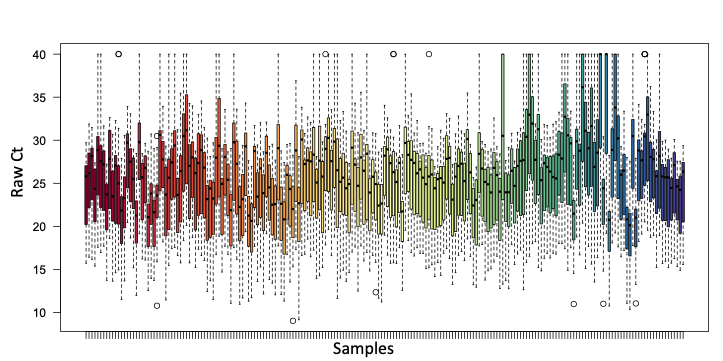


2.3. miRNA validation and models

2.3.1. Validation of miRNA candidates excluding mild-OSA.

e-Table 3: Validation of miRNA candidates excluding mild-OSA. There were 6 non-OSA patients reducing the power of this sub-analysis.

| miRNA | Fold change | p-value |
| --- | --- | --- |
| hsa-mir-181a | 0.315 | 0.002 |
| hsa-miR-133a | 0.324 | 0.054 |
| hsa-miR-345 | 0.556 | 0.081 |
| hsa-miR-495 | 0.441 | 0.086 |
| hsa-miR-199b | 0.522 | 0.139 |
| hsa-miR-340 | 0.620 | 0.198 |
| hsa-let-7d | 0.732 | 0.328 |
| hsa-miR-660 | 0.791 | 0.406 |
| hsa-miR-486-3p | 0.745 | 0.512 |
| hsa-miR-451 | 0.809 | 0.551 |
| hsa-miR-181a2 | 0.768 | 0.614 |
| hsa-miR-486 | 1.109 | 0.743 |
| hsa-miR-199a | 0.968 | 0.948 |
| hsa-miR-107 | 1.015 | 0.960 |

2.3.2. Validation of differentially expressed miRNAs in Women.

e-Table 4: Validation of miRNA candidates in women

| miRNA | Fold change | p-value | FDR correction |
| --- | --- | --- | --- |
| hsa-miR-451 | 1.641617132 | 0.057011678 | 0.53960381 |
| hsa-miR-199a | 3.968594886 | 0.127989504 | 0.53960381 |
| hsa-miR-340 | 2.114076774 | 0.185507751 | 0.53960381 |
| hsa-miR-107 | 2.596445604 | 0.205911237 | 0.53960381 |
| hsa-miR-133a | 2.079198266 | 0.212445111 | 0.53960381 |
| hsa-mir-181a | 1.78621407 | 0.290038965 | 0.53960381 |
| hsa-miR-181a2 | 2.380875818 | 0.290888298 | 0.53960381 |
| hsa-miR-486 | 1.286395732 | 0.308345035 | 0.53960381 |
| hsa-miR-199b | 1.847589946 | 0.380614767 | 0.592067416 |
| hsa-miR-486-3p | 1.454902206 | 0.433785555 | 0.596972855 |
| hsa-miR-495 | 1.827937146 | 0.4690501 | 0.596972855 |
| hsa-let-7d | 1.128385545 | 0.64411447 | 0.751466882 |
| hsa-miR-345 | 1.018070318 | 0.946577378 | 0.995775644 |
| hsa-miR-660 | 0.99700101 | 0.995775644 | 0.995775644 |

2.3.3. Univariate logistic regression analysis.

e-Table 5: Univariate logistic regression analysis of the miRNAs.

|  | **Estimate** | **SE** | **z-value** | **p-value** | **OR** | **LB (95% CI)** | **UB (95% CI)** |
| --- | --- | --- | --- | --- | --- | --- | --- |
| hsa-mir-181a | 1.37 | 0.39 | 3.53 | <0.001 | 3.92 | 1.87 | 8.59 |
| hsa-miR-199b | 1.96 | 0.63 | 3.11 | <0.001 | 7.12 | 2.38 | 30.75 |
| hsa-miR-345 | 1.37 | 0.39 | 3.53 | <0.001 | 3.92 | 1.87 | 8.59 |
| hsa-miR-133a | 1.3 | 0.4 | 3.27 | <0.001 | 3.68 | 1.73 | 8.34 |
| hsa-miR-340 | 1.34 | 0.46 | 2.95 | <0.001 | 3.83 | 1.65 | 10.06 |
| hsa-miR-486-3p | 1.32 | 0.52 | 2.56 | 0.01 | 3.75 | 1.47 | 11.58 |

2.4.4. Bioinformatic analysis of differentially expressed miRNAs.

e-Table 6: Pathways enrichment analysis.

| **Kegg Pathway** | **Number of genes** | **Benjamini** |
| --- | --- | --- |
| MAPK signaling pathway | 128 | 6,8E-5 |
| Sphingolipid signaling pathway | 67 | 4,3E-4 |
| Dilated cardiomyopathy | 50 | 6,1E-4 |
| Endocytosis | 124 | 4,6E-4 |
| Hypertrophic cardiomyopathy (HCM) | 47 | 4,7E-4 |
| Adrenergic signaling in cardiomyocytes | 76 | 8,7E-4 |
| Oxytocin signaling pathway | 81 | 7,9E-4 |
| Ras signaling pathway | 109 | 7,4E-4 |
| Renal cell carcinoma | 40 | 7,3E-4 |
| Pathways in cancer | 175 | 7,1E-4 |
| Insulin resistance | 58 | 1,8E-3 |
| Neurotrophin signaling pathway | 63 | 1,8E-3 |
| cAMP signaling pathway | 95 | 2,1E-3 |
| Retrograde endocannabinoid signaling | 54 | 3,0E-3 |
| Axon guidance | 65 | 2,9E-3 |
| Rap1 signaling pathway | 99 | 2,8E-3 |
| Calcium signaling pathway | 86 | 3,4E-3 |
| Insulin signaling pathway | 69 | 3,6E-3 |
| Fc epsilon RI signaling pathway | 39 | 3,6E-3 |
| Morphine addiction | 49 | 3,7E-3 |
| T cell receptor signaling pathway | 54 | 3,9E-3 |
| Cell adhesion molecules (CAMs) | 70 | 4,5E-3 |
| Cholinergic synapse | 57 | 4,7E-3 |
| \| Dopaminergic synapse \| \| --- \| | 64 | 4,8E-3 |
| Oocyte meiosis | 56 | 4,8E-3 |
| Glioma | 37 | 4,9E-3 |
| Regulation of actin cytoskeleton | 97 | 5,8E-3 |
| ErbB signaling pathway | 46 | 7,1E-3 |
| Osteoclast differentiation | 64 | 8,6E-3 |
| mTOR signaling pathway | 33 | 9,5E-3 |
| FoxO signaling pathway | 65 | 9,2E-3 |
| GnRH signaling pathway | 47 | 1,0E-2 |
| Signaling pathways regulating pluripotency of stem cells | 67 | 1,1E-2 |
| VEGF signaling pathway | 34 | 1,1E-2 |
| Leukocyte transendothelial migration | 58 | 1,1E-2 |
| Progesterone-mediated oocyte maturation | 45 | 1,1E-2 |
| Long-term potentiation | 36 | 1,2E-2 |
| Chemokine signaling pathway | 85 | 1,2E-2 |
| AMPK signaling pathway | 59 | 1,4E-2 |
| Hepatitis B | 68 | 1,6E-2 |
| Pancreatic cancer | 35 | 1,7E-2 |
| Non-small cell lung cancer | 31 | 1,8E-2 |
| Neuroactive ligand-receptor interaction | 119 | 1,8E-2 |
| Arrhythmogenic right ventricular cardiomyopathy (ARVC) | 37 | 2,3E-2 |
| Melanoma | 37 | 2,3E-2 |
| Inositol phosphate metabolism | 37 | 2,3E-2 |
| Prolactin signaling pathway | 37 | 2,3E-2 |
| HIF-1 signaling pathway | 48 | 2,5E-2 |
| Long-term depression | 32 | 2,9E-2 |
| Proteoglycans in cancer | 88 | 2,9E-2 |
| Glucagon signaling pathway | 48 | 3,0E-2 |
| Ubiquitin mediated proteolysis | 63 | 3,3E-2 |
| Cocaine addiction | 27 | 3,3E-2 |
| cGMP-PKG signaling pathway | 74 | 3,8E-2 |
| Wnt signaling pathway | 63 | 3,8E-2 |
| Gap junction | 43 | 3,8E-2 |
| Phosphatidylinositol signaling system | 47 | 3,8E-2 |
| Aldosterone synthesis and secretion | 40 | 4,0E-2 |
| Choline metabolism in cancer | 48 | 4,1E-2 |
| TNF signaling pathway | 50 | 4,1E-2 |
| Focal adhesion | 89 | 4,0E-2 |
| SNARE interactions in vesicular transport | 20 | 4,2E-2 |
| Thyroid hormone signaling pathway | 53 | 4,3E-2 |
| Estrogen signaling pathway | 47 | 4,2E-2 |
| Chronic myeloid leukemia | 36 | 4,3E-2 |
| NF-kappa B signaling pathway | 42 | 4,5E-2 |
| Adipocytokine signaling pathway | 35 | 4,7E-2 |
| Small cell lung cancer | 41 | 4,9E-2 |
| GABAergic synapse | 41 | 4,9E-2 |

e-Figure 6: Main biological processes regulated by the differentially expressed miRNAs.


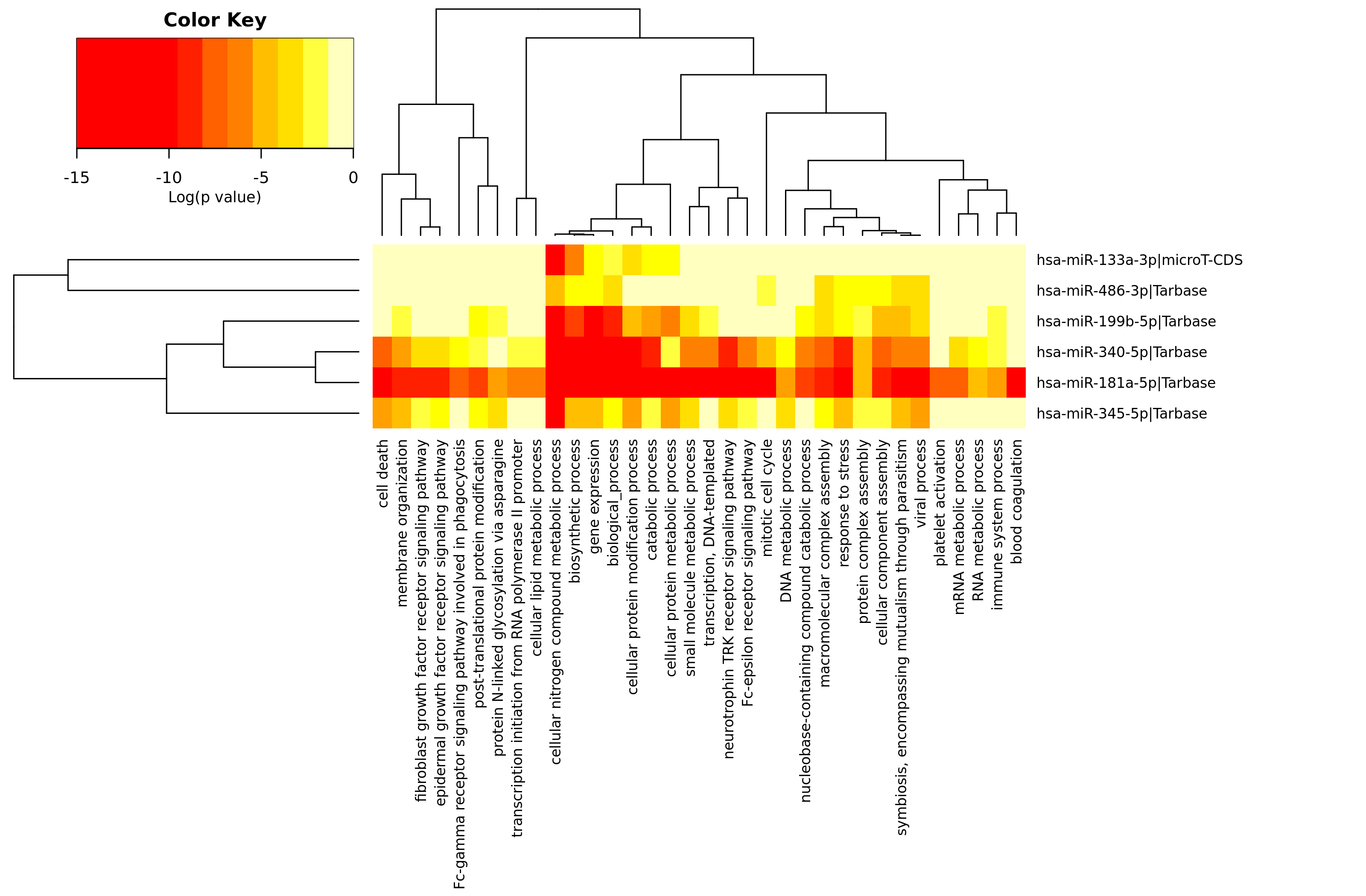

Supplement: Supplementary file 1 — Supplementary information [file 41598_2019_49940_MOESM1_ESM.docx]
